# Improved Estimation of Phenotypic Correlations Using Summary Association Statistics

**DOI:** 10.3389/fgene.2021.665252

**Published:** 2021-08-24

**Authors:** Ting Li, Zheng Ning, Xia Shen

**Affiliations:** ^1^Biostatistics Group, State Key Laboratory of Biocontrol, School of Life Sciences, Sun Yat-sen University, Guangzhou, China; ^2^Department of Medical Epidemiology and Biostatistics, Karolinska Institutet, Stockholm, Sweden; ^3^Centre for Global Health Research, Usher Institute, University of Edinburgh, Edinburgh, United Kingdom

**Keywords:** phenotypic correlation, genome-wide association, low MAF estimator, LD score regression, genetic correlation, minor allele frequency

## Abstract

Estimating the phenotypic correlations between complex traits and diseases based on their genome-wide association summary statistics has been a useful technique in genetic epidemiology and statistical genetics inference. Two state-of-the-art strategies, Z-score correlation across null-effect single nucleotide polymorphisms (SNPs) and LD score regression intercept, were widely applied to estimate phenotypic correlations. Here, we propose an improved Z-score correlation strategy based on SNPs with low minor allele frequencies (MAFs), and show how this simple strategy can correct the bias generated by the current methods. The low MAF estimator improves phenotypic correlation estimation, thus it is beneficial for methods and applications using phenotypic correlations inferred from summary association statistics.

## 1. Introduction

Phenotypic correlation is an essential parameter that helps understand observational correlations between complex traits and the etiological perspectives underlying complex diseases. Conventionally, estimation of the phenotypic correlation between a pair of phenotypes, by definition, is straightforward in a sample where both phenotypes are measured. Depending on the distribution of each phenotype, the estimated phenotypic correlation serves as a sufficient statistic for many linear statistical models, such as ordinary linear and logistic regressions, allowing us to assess parameters such as odds ratios of risk factors on disease outcomes.

Since a large number of genome-wide association studies (GWAS) were conducted, many GWASed phenotypes had measurements in an overlapping set of individuals, where many were from more than one participating cohort in GWAS meta-analysis. In practice, inference of the phenotypic correlations across these phenotypes would be complicated if estimating using the conventional way, which requires individual-level phenotypic data and subsequent meta-analysis. Fortunately, the phenotypic correlations can be estimated based on established GWAS summary statistics, especially when the proportion of sample overlap between two GWASed phenotypes is large. Two state-of-the-art strategies were proposed:

1. “*Z-cut” estimator*: The phenotypic correlation can be estimated by the correlation between the two sets of GWAS estimated effects or Z-scores, assuming the genetic effect per SNP is tiny or even null (Stephens, 2013; Zhu et al., 2015; Cichonska et al., 2016; Shen et al., 2017).
2. *LDSC intercept*. The phenotypic correlation can be estimated by the intercept of a bivariate linkage disequilibrium score regression (LDSC) (Bulik-Sullivan et al., 2015; Turley et al., 2018; Zheng et al., 2018).

Both estimators have reasonable performance in practice, however, bias exists for both strategies. Stephens (2013) reasoned that the correlation between Z-scores for the two phenotypes under the null is the same as the phenotypic correlation, thus “a set of putative null SNPs” were selected by taking SNPs with |*z*| <2. The same idea was also adopted by later studies (Zhu et al., 2015; Shen et al., 2017). The tool metaCCA (Cichonska et al., 2016) neglected the null effect requirement, as the genetic effect per variant is tiny, and computed the correlation between Z-scores across as many SNPs as possible. However, the Z-cut estimator can generate bias due to its constrain on the summary statistics of the SNPs (Zheng et al., 2018). LDSC intercept performs better and thus was adopted in statistical methods that requires pre-calculated phenotypic correlations (Turley et al., 2018; Zheng et al., 2018), but the intercept collects noise generated by population substructure, which may also lead to biased estimates of phenotypic correlations (Yengo et al., 2018).

Here, we revisit the correlation between GWAS summary statistics of two phenotypes and propose an alternative approach to select variants for the Z-score correlation estimation strategy. We show that selecting SNPs with low minor allele frequencies (MAFs) can lead to simple and consistent estimation of phenotypic correlations based on multi-SNP Z-score correlations. Via simulations, we show that the “low MAF” estimator can overcome bias generated by the Z-cut estimator and the LDSC intercept. With higher estimation efficiency, when applied to UK Biobank GWAS results, the low MAF estimator could discover 30% more significant phenotypic correlations than using the LDSC intercept.

## 2. Methods

We start by deriving a general mathematical form of the correlation between the summary statistics of two phenotypes *y*_1_ and *y*_2_, centered at a zero mean. The sample sizes for *y*_1_ and *y*_2_ are *N*_1_ and *N*_2_, respectively, and the overlapping part of *y*_1_ and *y*_2_ has a length of *N*_0_. For a single genetic variant in an association analysis, the model is *y*_*i*_ = *g*_*i*_β_*i*_+*e*_*i*_ (*i* = 1, 2), where *g*_*i*_ is the vector of genotypic values coded as 0, 1, and 2, and *e*_*i*_ are the residuals. Only β_*i*_ and *e*_*i*_ are random in the model. Assuming Hardy–Weinberg equilibrium (HWE), for SNP *j*, the genotypic values of *g*_*i*_ has a sample mean of 2*f*_*j*_ and a sample standard deviation of $\sqrt{2f_{j}\left(1-f_{j}\right)}$, where *f*_*j*_ is the allele frequency of the coding allele. *g*_1_ and *g*_2_ may differ due to different levels of sample overlap between the two phenotypes. At the single SNP *j* (omitted the subscripts *j* for simplicity),

(1) $$\begin{array}{r} \left(\begin{array}{c} \beta_{1}\\ \beta_{2} \end{array}\right)\sim \text{dist}\left(0,\left[\begin{array}{cc} \sigma_{\beta_{1}}^{2} & r_{G}\sigma_{\beta_{1}}\sigma_{\beta_{2}}\\ r_{G}\sigma_{\beta_{1}}\sigma_{\beta_{2}} & \sigma_{\beta_{2}}^{2} \end{array}\right]\right), \end{array}$$

and

(2) $$\begin{array}{r} \left(\begin{array}{c} e_{1}\\ e_{2} \end{array}\right)\sim \text{dist}\left(0,\left[\begin{array}{cc} \sigma_{1}^{2}I_{N_{1}\times N_{1}} & r_{E}\sigma_{1}\sigma_{2}\left(\begin{array}{cc} I_{N_{0}\times N_{0}}\\ \; & 0_{N_{1}^{\prime }\times N_{2}^{\prime }} \end{array}\right)\\ r_{E}\sigma_{1}\sigma_{2}\left(\begin{array}{cc} I_{N_{0}\times N_{0}}\\ \; & 0_{N_{2}^{\prime }\times N_{1}^{\prime }} \end{array}\right) & \sigma_{2}^{2}I_{N_{2}\times N_{2}} \end{array}\right]\right), \end{array}$$

where $N_{i}^{\prime }=N_{i}-N_{0}$, *r*_*G*_ is the underlying genetic correlation at SNP *j*, and *r*_*E*_ is the residual correlation. dist() denotes a multivariate distribution with a given mean vector and a variance–covariance matrix. In an association study, *r*_*G*_ is un-identifiable at a single SNP. The estimated genetic effects are ${\hat{\beta }}_{i}=g_{i}^{\prime }y_{i}/g_{i}^{\prime }g_{i}$, then

(3) $$\begin{array}{l} \text{var}\left({\hat{\beta }}_{i}\right)=\frac{\text{var}\left(g_{i}^{\prime }y_{i}\right)}{{\left(g_{i}^{\prime }g_{i}\right)}^{2}}\\ \;=\frac{\text{var}\left(g_{i}^{\prime }g_{i}\beta_{i}+g_{i}^{\prime }e_{i}\right)}{{\left(g_{i}^{\prime }g_{i}\right)}^{2}}\\ \;=\sigma_{\beta_{i}}^{2}+\sigma_{i}^{2}{\left(g_{i}^{\prime }g_{i}\right)}^{-1}. \end{array}$$

Denote *g* as the overlapping part of *g*_1_ and *g*_2_, and let *x* and *z* be the rest parts of *g*_1_ and *g*_2_, respectively. We have $g_{i}^{\prime }g_{i}\approx 2f\left(1-f\right)N_{i}$ (*i* = 1, 2) and $g^{\prime }g\approx 2f\left(1-f\right)N_{0}$. So that, defining $z_{i}={\hat{\beta }}_{i}/\sqrt{\text{var}\left({\hat{\beta }}_{i}\right)}$, we have

(4) $$\begin{array}{l} \text{cor}\left({\hat{\beta }}_{1},{\hat{\beta }}_{2}\right)\\ =\text{cor}\left(z_{1},z_{2}\right)\\ =\frac{\text{cov}\left(g_{1}^{\prime }y_{1},g_{2}^{\prime }y_{2}\right)}{\sqrt{{\left(g_{1}^{\prime }g_{1}\right)}^{2}\sigma_{\beta_{1}}^{2}+g_{1}^{\prime }g_{1}\sigma_{1}^{2}}\sqrt{{\left(g_{2}^{\prime }g_{2}\right)}^{2}\sigma_{\beta_{2}}^{2}+g_{2}^{\prime }g_{2}\sigma_{2}^{2}}}\\ =\frac{\left(g_{1}^{\prime }g_{1}\right)\left(g_{2}^{\prime }g_{2}\right)\text{cov}\left(\beta_{1},\beta_{2}\right)+g_{1}^{\prime }\text{cov}\left(e_{1},e_{2}\right)g_{2}}{\sqrt{{\left(g_{1}^{\prime }g_{1}\right)}^{2}\sigma_{\beta_{1}}^{2}+g_{1}^{\prime }g_{1}\sigma_{1}^{2}}\sqrt{{\left(g_{2}^{\prime }g_{2}\right)}^{2}\sigma_{\beta_{2}}^{2}+g_{2}^{\prime }g_{2}\sigma_{2}^{2}}}\\ =\frac{\left(g_{1}^{\prime }g_{1}\right)\left(g_{2}^{\prime }g_{2}\right)r_{G}\sigma_{\beta_{1}}\sigma_{\beta_{2}}+r_{E}\sigma_{1}\sigma_{2}\left(g^{\prime },x^{\prime }\right)\left(\begin{array}{cc} I_{N_{0}\times N_{0}}\\ \; & 0_{N_{1}^{\prime }\times N_{2}^{\prime }} \end{array}\right)\left(\begin{array}{c} g\\ z \end{array}\right)}{\sqrt{{\left(g_{1}^{\prime }g_{1}\right)}^{2}\sigma_{\beta_{1}}^{2}+g_{1}^{\prime }g_{1}\sigma_{1}^{2}}\sqrt{{\left(g_{2}^{\prime }g_{2}\right)}^{2}\sigma_{\beta_{2}}^{2}+g_{2}^{\prime }g_{2}\sigma_{2}^{2}}}\\ =\frac{\left(g_{1}^{\prime }g_{1}\right)\left(g_{2}^{\prime }g_{2}\right)r_{G}\sigma_{\beta_{1}}\sigma_{\beta_{2}}+g^{\prime }gr_{E}\sigma_{1}\sigma_{2}}{\sqrt{{\left(g_{1}^{\prime }g_{1}\right)}^{2}\sigma_{\beta_{1}}^{2}+g_{1}^{\prime }g_{1}\sigma_{1}^{2}}\sqrt{{\left(g_{2}^{\prime }g_{2}\right)}^{2}\sigma_{\beta_{2}}^{2}+g_{2}^{\prime }g_{2}\sigma_{2}^{2}}}\\ =\frac{2f\left(1-f\right)\sqrt{N_{1}N_{2}}r_{G}\sigma_{\beta_{1}}\sigma_{\beta_{2}}+N_{0}/\sqrt{N_{1}N_{2}}r_{E}\sigma_{1}\sigma_{2}}{\sqrt{2f\left(1-f\right)N_{1}\sigma_{\beta_{1}}^{2}+\sigma_{1}^{2}}\sqrt{2f\left(1-f\right)N_{2}\sigma_{\beta_{2}}^{2}+\sigma_{2}^{2}}}, \end{array}$$

When σ_β_*i*__ = 0 (*i* = 1, 2), i.e., for any variant with null genetic effect, the above equation simplifies to

(5) $$\begin{array}{r} \text{cor}\left({\hat{\beta }}_{1},{\hat{\beta }}_{2}\right)=\text{cor}\left(z_{1},z_{2}\right)=\frac{N_{0}}{\sqrt{N_{1}N_{2}}}r_{E}=\frac{N_{0}}{\sqrt{N_{1}N_{2}}}r\left(y_{1},y_{2}\right) \end{array}$$

where *r*(*y*_1_, *y*_2_) is the phenotypic correlation based on completely overlapped individual-level data. Thus, in order to estimate *r*(*y*_1_, *y*_2_), we can estimate cor(*z*_1_, *z*_2_) instead. Particularly, for perfectly overlap samples, i.e., *N*_0_ = *N*_1_ = *N*_2_, we have cor(*z*_1_, *z*_2_) = *r*(*y*_1_, *y*_2_), which is the phenotypic correlation estimator derived by Zhu et al. (Zhu et al., 2015). Our theory above in Equation (4) is an extension of Zhu et al.'s theory, covering the substantial amount of genetic correlation across the genome. Only when σ_β_*i*__ or *f* is zero and the samples perfectly overlap between the two traits, Equation (4) reduces to Zhu et al.'s result. Equation (4) shows the reasoning behind the low MAF estimator, i.e., in practice, one can hardly control σ_β_*i*__ but *f* to be close to zero, so that the correlation between Z-scores becomes close to the phenotypic correlation, subject to a shrinkage factor if the samples do not perfectly overlap.

The result suggests that the phenotypic correlation between the two phenotypes *y*_1_ and *y*_2_, subject to a shrinkage factor corresponding to sample overlap, can be estimated by the sample correlation of the summary statistics across any sufficient number of null variants. This leads to a commonly adopted strategy of estimating the phenotypic correlation from summary association statistics by taking a subset with, e.g., |*z*_*i*_| <2 (*i* = 1, 2). However, we will show that such thresholding may introduce bias into the correlation estimate.

According to Equation (4), null genetic effect for the variant is a sufficient but not necessary condition for cor(*z*_1_, *z*_2_) to reduce to Equation (5). When *f* = 0, Equation (4) also becomes (5). In practice, the phenotypic correlation can be estimated by the correlation of the summary statistics across a sufficient number of variants with very low MAFs, *regardless* of whether the genetic effects are null. The thresholding on the MAF does not directly introduce a threshold on β_*i*_ or *z*_*i*_ so that not prone to bias in the phenotypic correlation estimation.

### 2.1. Simulation Settings

We conducted a series of simulations to compare the low MAF estimators with the Z-cut estimators. Based on the real UK Biobank genotypes, two phenotypes were simulated based on the 784,256 genotyped SNPs in the UK Biobank and the model:

(6) $$\begin{array}{r} y_{i}=X_{i}\beta_{i}+e_{i} \end{array}$$

where *i* = 1, 2 is the phenotype index, *X*_*i*_ is the matrix of genotypic values, **β**_*i*_ is the vector of genetic effects, and *e*_*i*_ are the residuals. Each column of *X*_*i*_ was standardized to have a zero mean and unit variance. Two heritability values (*h*^2^) for the phenotypes were considered: 0.3 and 0.6. The genetic effects and residuals were drawn from a Gaussian distribution with corresponding variance components: $\beta_{i}\sim N\left(0,\left(h^{2}/M\right)I\right)$, where *M* represents the number of causal variants, and $e_{i}\sim N\left(0,\left(1-h^{2}\right)I\right)$. Each phenotype was simulated for 168,000 genomic British individuals. Two different scenarios of the proportion of causal SNPs were considered: 10% randomly selected SNPs and 100%. Three scenarios of the true genetic correlation (correlation between the **β**_*i*_ vectors) were considered: 0, 0.5, and 1. Three scenarios of sample overlap proportions were considered: 0 (no overlap), 0.5 (half overlapped), and 1 (perfectly matched).

Nine different methods for phenotypic correlation estimation were considered, including the true phenotypic correlation estimator was based on the individual-level phenotype data and the other eight that use the correlation between Z-scores for the estimation. For the illustration purpose, an estimator based on the Z-scores of 500 simulated SNPs with random genotypes and zero genetic effects was considered (referred to as “random” estimator here on). For the low MAF and Z-cut estimators, without loss of generality, Z-scores of the 12,966 SNPs on chromosome 22 were used for the estimation. Four MAF cutoffs for the low MAF estimator, 0.5 (all SNPs), 0.05, 0.005, and 0.0005, were considered. Three absolute value cutoffs for the Z-cut method, 0.5, 1, and 2, were considered.

For each method, the number of SNPs used for estimation in the simulation is given in Supplementary Table 3. Each scenario of the simulation was repeated for 30 times. The estimates were saved for evaluating the consistency of the phenotypic correlation estimators and their corresponding standard errors. All the above simulation analyses were performed using the R language (version 3.6.3).

In order to compare the low MAF estimator with the LDSC-intercept estimator, we conducted another simulation that used the real UK Biobank genotypes for 336,000 genomic British individuals across the 1,029,876 quality-controlled HapMap3 SNPs selected by the high-definition likelihood (HDL) software (Ning et al., 2020). Similar to the above, we draw the genetic effects across 10% of these SNPs from a normal distribution with zero mean. The heritability, phenotypic, genetic, and residual correlations all had a true value of 0.5. GWAS Z-scores of 70,042 SNPs with MAF <5 × 10^−4^ were used for the low MAF estimator. Two reference panels were evaluated for LDSC, including the ldsc software inbuilt 1000 Genomes reference and the UK Biobank reference based on the HDL software reference data.

## 3. Results

### 3.1. The Low MAF Estimator Corrects the Bias of the Z-Cut Estimator

**Figure 1:**
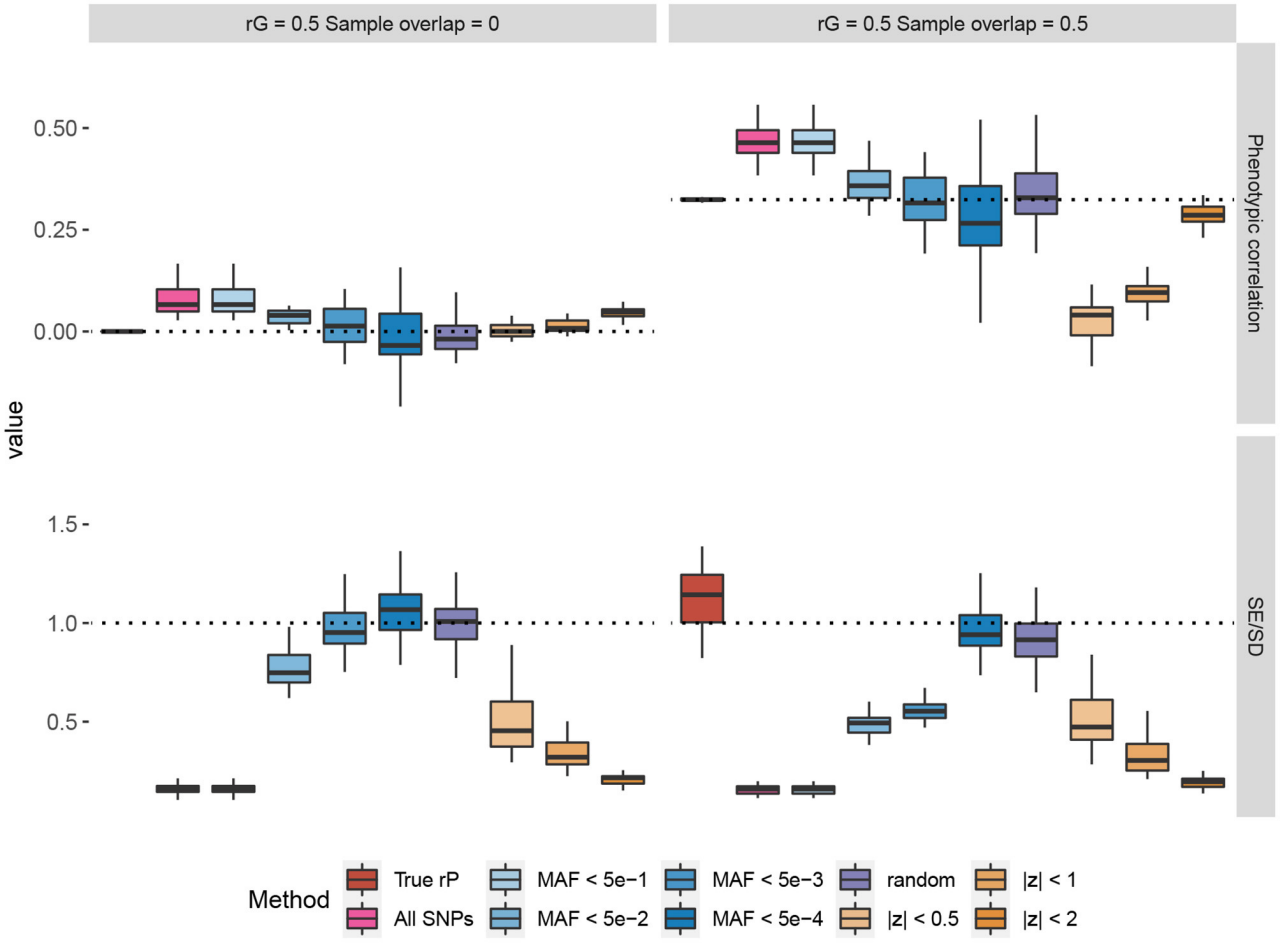
Simulations comparing the Z-cut and low minor allele frequency (MAF) estimators for phenotypic correlation. The box plots show the simulation results from 30 replicates, where in each replicate, two phenotypes with heritability of 0.3 were simulated based on 10% randomly selected causal single nucleotide polymorphisms (SNPs) from the 784,256 genotyped SNPs in the UK Biobank. The true genetic correlation (rG) were set to 0.5 and the residual correlations were set to 0.25 for each pair of traits. Two scenarios of sample overlap proportions are shown. In the top panels, each phenotype was simulated for 168,000 genomic British individuals. The box plots compare the estimated phenotypic correlations using different estimators. The dash lines represent the true values. The bottom panels compare the estimated standard errors (SE) across the replicates to the standard deviation (SD) of the phenotypic correlation estimates across the 30 replicates, and each phenotype was simulated for 1,000 genomic British individuals. The dash lines at 1 represent that the estimated SE matches the empirical sampling SD. The “random” method represents the estimator based on 500 simulated SNPs with random genotypes and zero genetic effects. The true phenotypic correlation (rP) estimator was based on the individual-level phenotype data. The other estimators were based on the SNPs on chromosome 22 (numbers given in Supplementary Table 3).

In Figure 1, we provide some representative simulation results when the genetic correlation between the traits is 0.5. The general conclusion is that the low MAF estimator with a low enough MAF cutoff is able to overcome the bias of the Z-cut estimator. The complete simulation results comparing the low MAF and Z-cut estimators were summarized and given in Supplementary Figures 1–8 and Supplementary Tables 1, 2. Here, we summarize the key points as follows.

- When the samples do not overlap between the two traits, the phenotypic correlation is by definition zero. When no genetic correlation exists, all the methods that use the correlation between Z-scores give consistent zero estimates for the phenotypic correlation. However, bias in the estimation could happen when the genetic correlation is non-zero, which agrees with our theory in section 2. When there is a non-zero genetic correlation spread across the genome, only those methods that use the SNPs capturing little genetic variance would yield a consistent estimate for the phenotypic correlation, e.g., the random estimator where the SNPs capture absolutely zero genetic variance and the low MAF estimator with low enough MAF cutoffs.
- In overlapping samples, when the genetic correlation is zero, slight bias can be observed when using the Z-scores of common SNPs for phenotypic correlation estimation. Such bias can be corrected when a sufficiently low MAF cutoff is applied to the low MAF estimator. In partially overlapping scenarios, the observational phenotypic correlation in individual-level data can be estimated by adjusting the shrinkage factor $N_{0}/\sqrt{N_{1}N_{2}}$.
- For the UK Biobank real genotype data, a 0.005 cutoff is low enough to yield a consistent estimate of the phenotypic correlation. Nevertheless, unless too few SNPs exist, using SNPs with MAF <5 × 10^−4^ is recommended, as the standard error of the estimator can be consistently obtained in a simple way. The LD between low MAF SNPs is so small that we may consider the SNP genotypes as independent. Therefore, when simply obtaining the test statistic for the Pearson's correlation coefficient, the standard error for the Wald test can be back-calculated from the nominal p-value. The simulation results showed that the SEs of the low MAF method calculated in this way are consistent with the empirical standard deviation across the simulation repeats.
- Applying a low MAF cutoff on the pre-filtered SNPs based on the Z-cut method could reduce the bias in some cases, but the bias cannot be completely overcome as the Z-cut method itself is a biased sampling strategy of the SNPs.

### 3.2. The Low MAF Estimator Corrects the Bias of the LDSC Intercept Estimator

**Figure 2:**
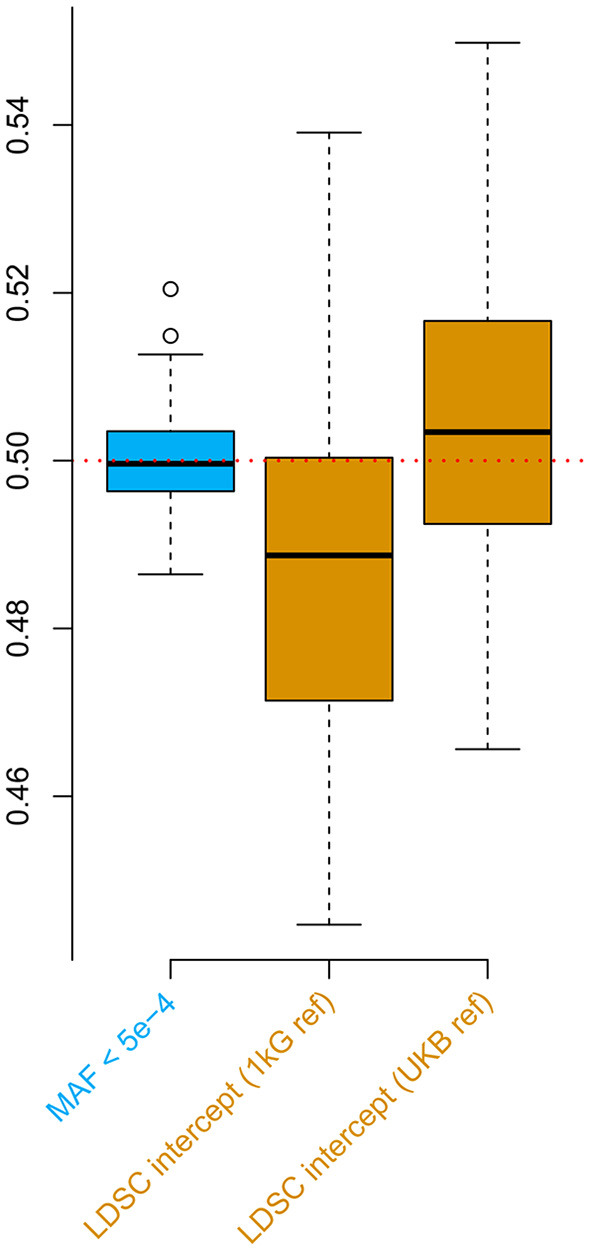
Simulations comparing the low minor allele frequency (MAF) estimator and LD score regression (LDSC) intercept using the UK Biobank genotype data. The box plots show the results from 100 replicates, where in each replicate, two phenotypes with heritability of 0.5 were simulated for 336,000 genomic British individuals. The true phenotypic, genetic, and residual correlations were all set to 0.5. The low MAF estimates were based on 70,042 single nucleotide polymorphisms (SNPs) with MAF < 5 × 10^−4^. 1 kG ref: LD scores calculated based on the 1000 Genomes reference panel; UKB ref: LD scores calculated based on the UK Biobank reference panel.

For the second simulation, we observed downward bias in the LDSC intercept when the default 1000 Genomes reference was applied (Figure 2). Such a bias was overcome by the UK Biobank reference, nevertheless, the estimates were slightly inflated possibly due to the population substructure in the UKB genomic British individuals (Yengo et al., 2018). These biases were all absent when applying the low MAF estimator for the phenotypic correlation. Furthermore, the low MAF estimator had a substantially higher estimation efficiency than the LDSC intercept (Supplementary Table 4).

### 3.3. Example Based on UK Biobank GWAS Summary Statistics

**Figure 3:**
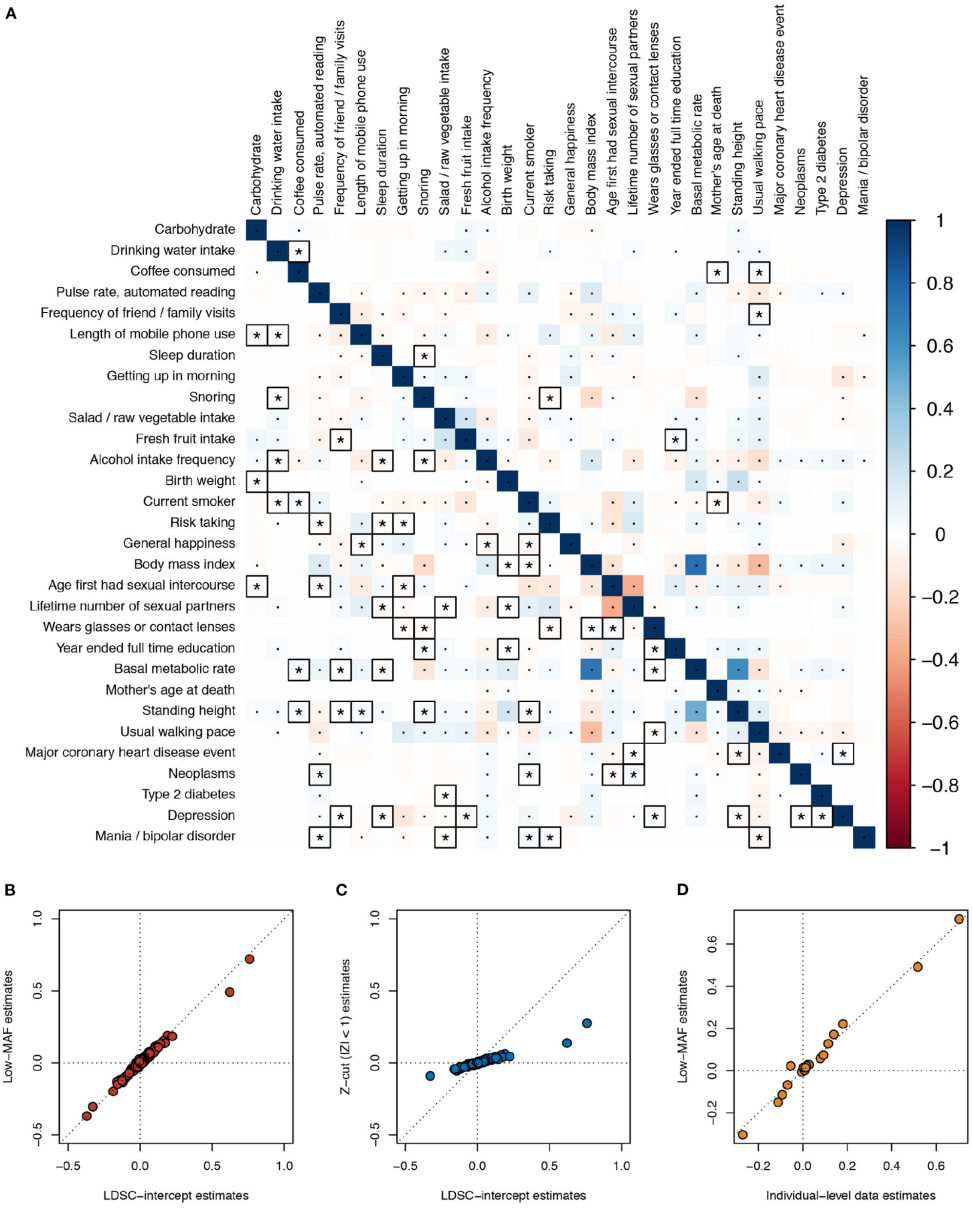
Phenotypic correlation estimates across 30 UK Biobank traits. **(A)** Estimates using the low minor allele frequency (MAF) estimator (lower triangle) and LD score regression (LDSC) intercept (upper triangle) are compared. The low MAF estimates were based on 70,042 single nucleotide polymorphisms (SNPs) with MAF < 5 × 10^−4^. The default 1000 Genomes reference panel was used in LDSC. Bonferroni-corrected significant correlations with *P* < 0.05/435 are marked with asterisks or dots, where those correlations that are only significant using one of the two methods are marked with asterisks and squares. **(B)** Scatterplot comparing the LDSC intercept and low MAF estimates. **(C)** Scatterplot comparing the LDSC intercept and the Z-cut (|*Z*| < 1) estimates. **(D)** Scatterplot comparing the individual-level observational data and low MAF estimates; for the seven traits, we have data for, i.e., body mass index, basal metabolic rate, usual walking pace, standing height, birth weight, coffee consumed, and year ended full time education.

As a real data example, we applied the different estimation methods on the same 30 UK Biobank phenotypes used in Ning et al.'s study in genetic correlation estimation (Ning et al., 2020), where the GWAS summary statistics are publicly available (see Data Availability Statement section). The low MAF estimates were based on 70,042 SNPs with MAF <5 × 10^−4^, and the LD scores were calculated based on the 1000 Genomes reference panel (default). At a 5% Bonferroni-corrected p-value threshold for 435 pairs of traits, the low MAF method discovered 223 significant phenotypic correlations, and LDSC intercept discovered 171. Among these, 61 phenotypic correlations were only significant in the low MAF method, vs. 9 only significant using the LDSC intercept (Figure 3A). The point estimates of the phenotypic correlations by the low MAF method and bivariate LDSC intercept were nearly the same (Figure 3B). As expected, when a Z-cut method is applied, the estimates became severely biased toward zero (Figure 3C). For seven of these phenotypes that we have individual-level data in our UK Biobank project (No. 14302), including body mass index, basal metabolic rate, usual walking pace, standing height, birth weight, coffee consumed, and year ended full time education, we extracted the initial measurement values. In order to be more consistent with the GWAS quality control procedure, we took away the effects of sex and age on these phenotypes by taking the residuals from linear regressions. The residuals were subsequently inverse-Gaussian transformed. After computing the individual-level observational phenotypic correlations and adjusted for the shrinkage factor $N_{0}/\sqrt{N_{1}N_{2}}$, the estimates were close to the low MAF estimates for these 21 pairs of traits (Figure 3D).

## 4. Discussion

We have proposed the low MAF estimator of phenotypic correlations based on GWAS summary statistics, as an improvement of the Z-score correlation strategy based on all SNPs or SNPs that pass a particular Z-score cutoff. The estimator overcomes the bias generated when thresholding on summary association statistics and even that generated in the bivariate LDSC intercept. We suggest the use of the low MAF phenotypic correlation estimator in future practice. The more consistent and efficient estimation can improve our understanding of connections across human complex traits and diseases.

Although the low MAF method also introduces a filter on the tested SNPs, it is a threshold-free technique for the genetic effect parameter. Thus, the low MAF estimator does not constrain the estimated genetic effects of selected SNPs, equivalent to sampling a set of null effect SNPs from the genome. This explains why “putative null effect” SNPs with, e.g., |*z*| <2 generate bias, whereas the low MAF estimator does not. Even if all the SNPs are null, some of them will generate z-score with |*z*|>2 due to randomness. Removing them would lead to bias.

As the low MAF estimator is equivalent to sampling a set of null effect SNPs from the genome, the resulted phenotypic correlation estimates are close to those estimated using individual-level phenotypic data. In the real UKB genotype data simulation, we showed that the LDSC intercept could not produce consistent estimates of the phenotypic correlation due to population substructure. Such a complication in LDSC was overcome by the low MAF estimator; although the GWAS summary statistics were used, the estimator approximates observed phenotypic correlation and is irrelevant to genetic data structure. For example, the genotypic data are treated as nuisance in the low MAF estimator.

As a comparative reference, we considered the “random” estimator using the Z-scores of 500 completely “irrelavant” SNPs. These SNPs were simply randomly generated, with random genotypes and zero genetic effects. These “bad” SNPs in GWAS appeared to be perfect for estimating the phenotypic correlation. The reason is simple according to our theory: they explain no phenotypic variance, so correlating their Z-scores for two traits becomes equivalent to correlating the phenotypic values themselves. This also explains why using the low MAF SNPs almost does the same: the low MAF SNPs explain little phenotypic variance. As LD between low MAF SNPs is rather low, using the low MAF SNPs is also helpful for getting the standard errors of the phenotypic correlation estimates. In the real data, low MAF SNPs are usually prone to genotyping errors or imputation failures if imputed. For the phenotypic correlation estimation purpose, even such errors are good, as they add more noise to the genotype data so that the SNP genotypes are even closer to noise.

Different sample overlap scenarios can be adjusted to obtain a consistent estimate of the observational phenotypic correlation. As long as *N*_0_, *N*_1_, and *N*_2_ are known, the shrinkage factor $N_{0}/\sqrt{N_{1}N_{2}}$ can be adjusted in the low MAF estimator. It should be noted that the adjustment becomes bad when *N*_0_ is too small. In the extreme case, when *N*_0_ = 0, i.e., in non-overlapping samples, there is no information we can learn about the phenotypic correlation from the two sets of GWAS summary statistics.

For binary phenotypes, an advantage of summary-statistics-based estimators, such as the low MAF estimator, is that it estimates the underlying phenotypic correlations on the liability scale. The liabilities follow an unobservable logistic distribution therefore the estimates are not exactly the same as the observed phenotypic correlations directly computed using the 0–1 outcome data. The phenotypic correlation estimates on the liability scale is mathematically easier to interpret and can be transformed into odds ratios from logistic regressions. Although for low MAF SNPs (rare variants) the GWAS test statistics would be generally inflated when the case–control data are unbalanced (Ma et al., 2013), the correlation between the Z-scores of two traits across the genome is still a valid estimator for the phenotypic correlation, which is not affected by low allele frequencies (Supplementary Figure 9).

## Data Availability Statement

The individual-level genotype and phenotype data are available by application from the UK Biobank (http://www.ukbiobank.ac.uk/). The UKBB GWAS summary statistics by the Neale's lab can be obtained from http://www.nealelab.is/uk-biobank/.

## Author Contributions

XS initiated and coordinated the study and drafted the manuscript. TL and ZN contributed to data analysis. All authors contributed to manuscript writing and gave final approval to publish.

## Conflict of Interest

The authors declare that the research was conducted in the absence of any commercial or financial relationships that could be construed as a potential conflict of interest.

## Publisher's Note

All claims expressed in this article are solely those of the authors and do not necessarily represent those of their affiliated organizations, or those of the publisher, the editors and the reviewers. Any product that may be evaluated in this article, or claim that may be made by its manufacturer, is not guaranteed or endorsed by the publisher.
