# Supplementary material for: Improved Estimation of Phenotypic Correlations Using Summary Association Statistics
Source: Front Genet. 2021 Aug 24;12:665252. doi: 10.3389/fgene.2021.665252 (PMC8421683; doi:10.3389/fgene.2021.665252)
Supplement: Supplementary file 1 [file Data_Sheet_1.PDF]

Supplementary Material

for

Improved estimation of phenotypic correlations using summary association statistics

by Ting Li, Zheng Ning, Xia Shen

Supplementary Figures

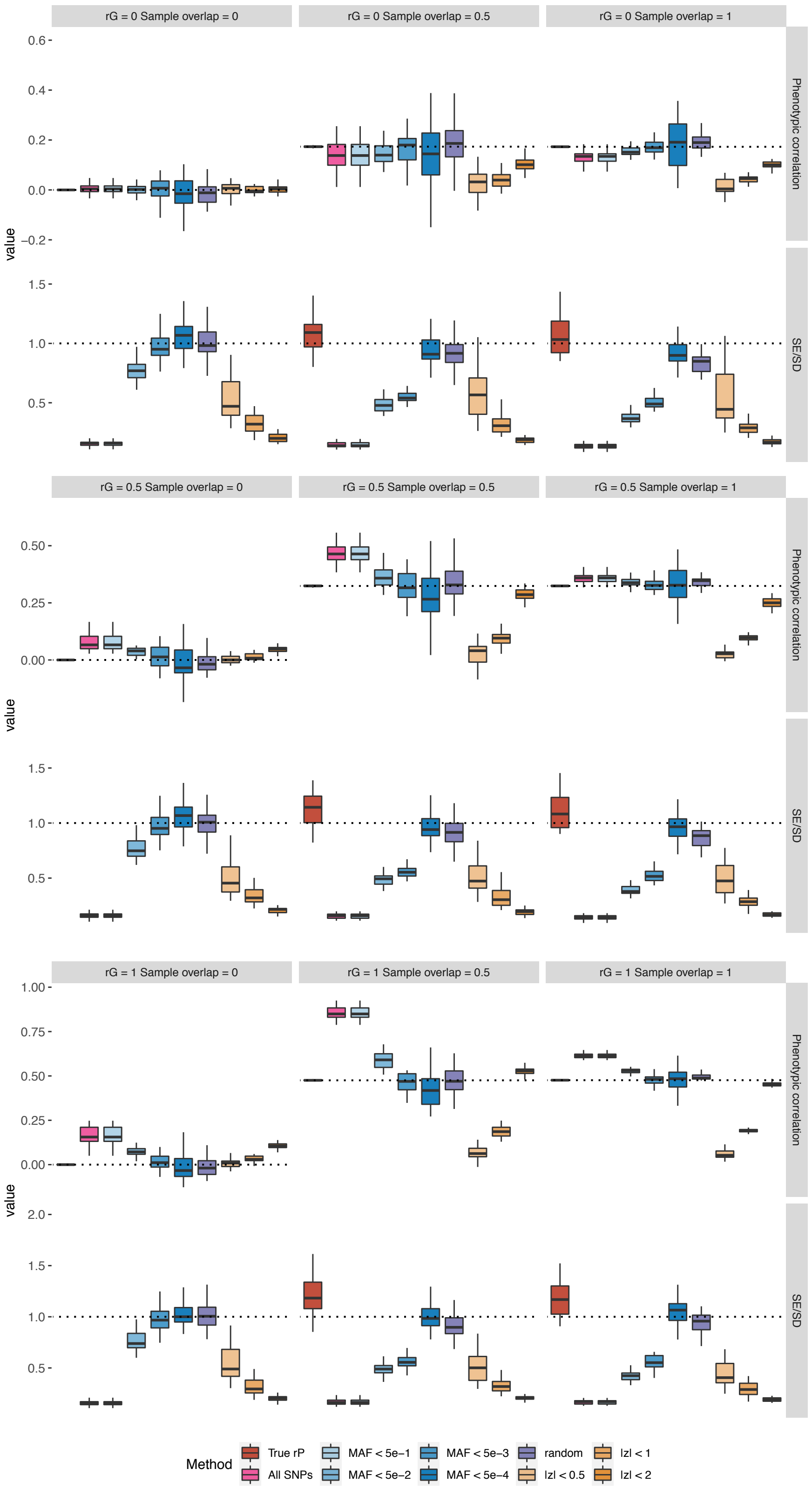

Supplementary Figure 1: Simulations comparing the Z-cut and low-MAF estimators for phenotypic correlation of simulated traits with the heritability of 0.3 based on 10% casual variants. The box plots show the simulation results from 30 replicates, where in each replicate, two phenotypes with heritability of 0.3 were simulated based on 10% randomly selected causal SNPs from the 784,256 genotyped SNPs in the UK Biobank. The true genetic correlation (rG) were set to 0,0.5,1 and the residual correlations were set to 0.25 for each pair of traits. Three scenarios of sample overlap proportions are shown. In the top panels, each phenotype was simulated for 168,000 genomic British individuals. The box plots compare the estimated phenotypic correlations using different estimators. The dash lines represent the true values. The bottom panels compare the estimated standard errors (SE) across the replicates to the standard deviation (SD) of the phenotypic correlation estimates across the 30 replicates, and each phenotype was simulated for 1,000 genomic British individuals. The dash lines at 1 represent that the estimated SE matches the empirical sampling SD. The "random" method represents the estimator based on 500 simulated SNPs with random genotypes and zero genetic effects. The true phenotypic correlation (rP) estimator was based on the individual-level phenotype data. The other estimators were based on the 12,966 SNPs on chromosome 22. MAF: minor allele frequency.

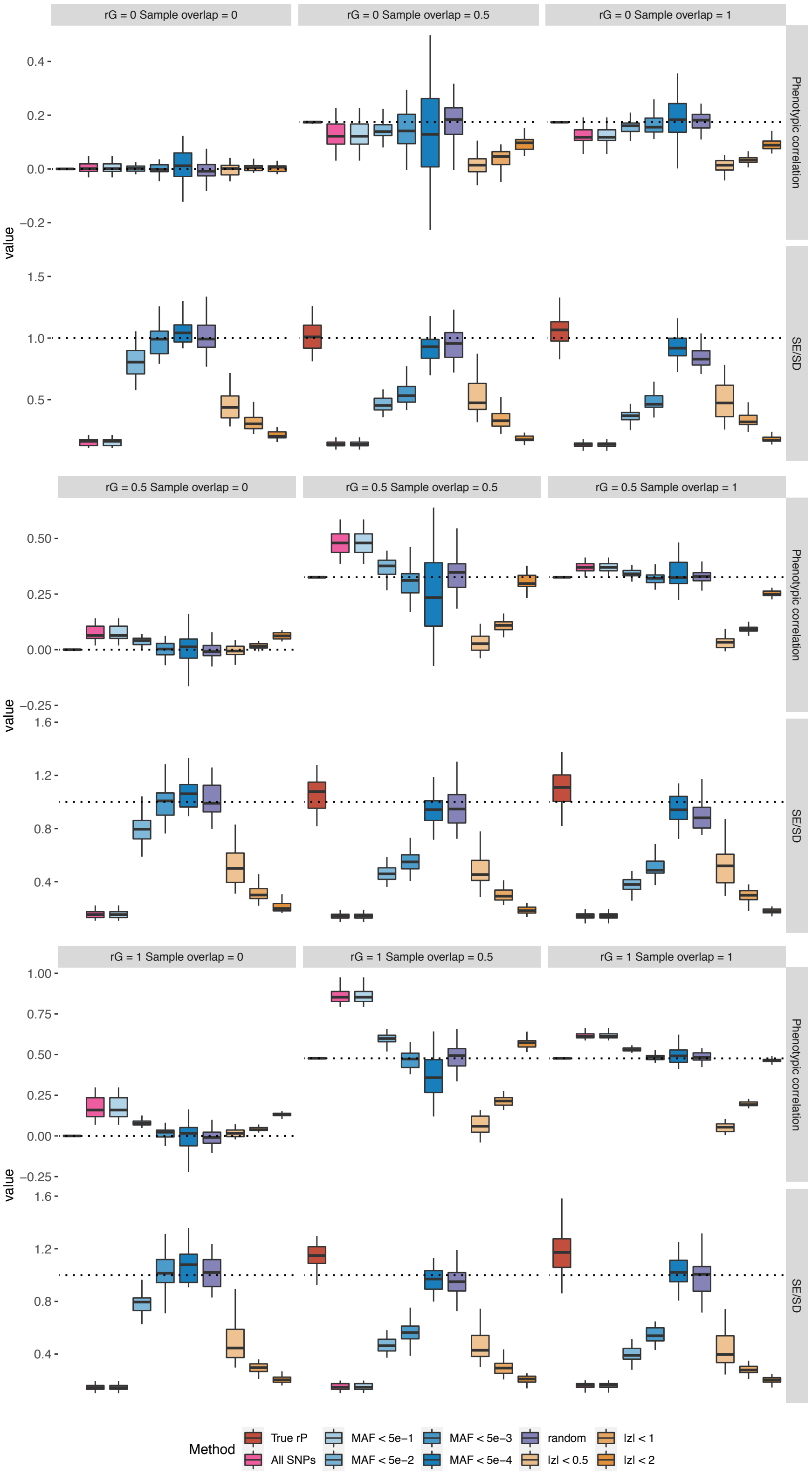
rG = 0 Sample overlap = 0
rG = 0 Sample overlap = 0.5
rG = 0 Sample overlap = 1
rG = 0.5 Sample overlap = 0
rG = 0.5 Sample overlap = 0.5
rG = 0.5 Sample overlap = 1
rG = 1 Sample overlap = 0
rG = 1 Sample overlap = 0.5
rG = 1 Sample overlap = 1
Phenotypic correlation
SE/SD
value
Method
True rP
All SNPs
MAF < 5e−1
MAF < 5e−2
MAF < 5e−3
MAF < 5e−4
random
|z| < 0.5
|z| < 1
|z| < 2

Supplementary Figure 2: Simulations comparing the Z-cut and low-MAF estimators for phenotypic correlation of simulated traits with the heritability of 0.3 based on 100% casual variants. The box plots show the simulation results from 30 replicates, where in each replicate, two phenotypes with heritability of 0.3 were simulated based on 100% causal SNPs from the 784,256 genotyped SNPs in the UK Biobank. The true genetic correlation (rG) were set to 0,0.5,1 and the residual correlations were set to 0.25 for each pair of traits. Three scenarios of sample overlap proportions are shown. In the top panels, each phenotype was simulated for 168,000 genomic British individuals. The box plots compare the estimated phenotypic correlations using different estimators. The dash lines represent the true values. The bottom panels compare the estimated standard errors (SE) across the replicates to the standard deviation (SD) of the phenotypic correlation estimates across the 30 replicates, and each phenotype was simulated for 1,000 genomic British individuals. The dash lines at 1 represent that the estimated SE matches the empirical sampling SD. The "random" method represents the estimator based on 500 simulated SNPs with random genotypes and zero genetic effects. The true phenotypic correlation (rP) estimator was based on the individual-level phenotype data. The other estimators were based on the 12,966 SNPs on chromosome 22. MAF: minor allele frequency.

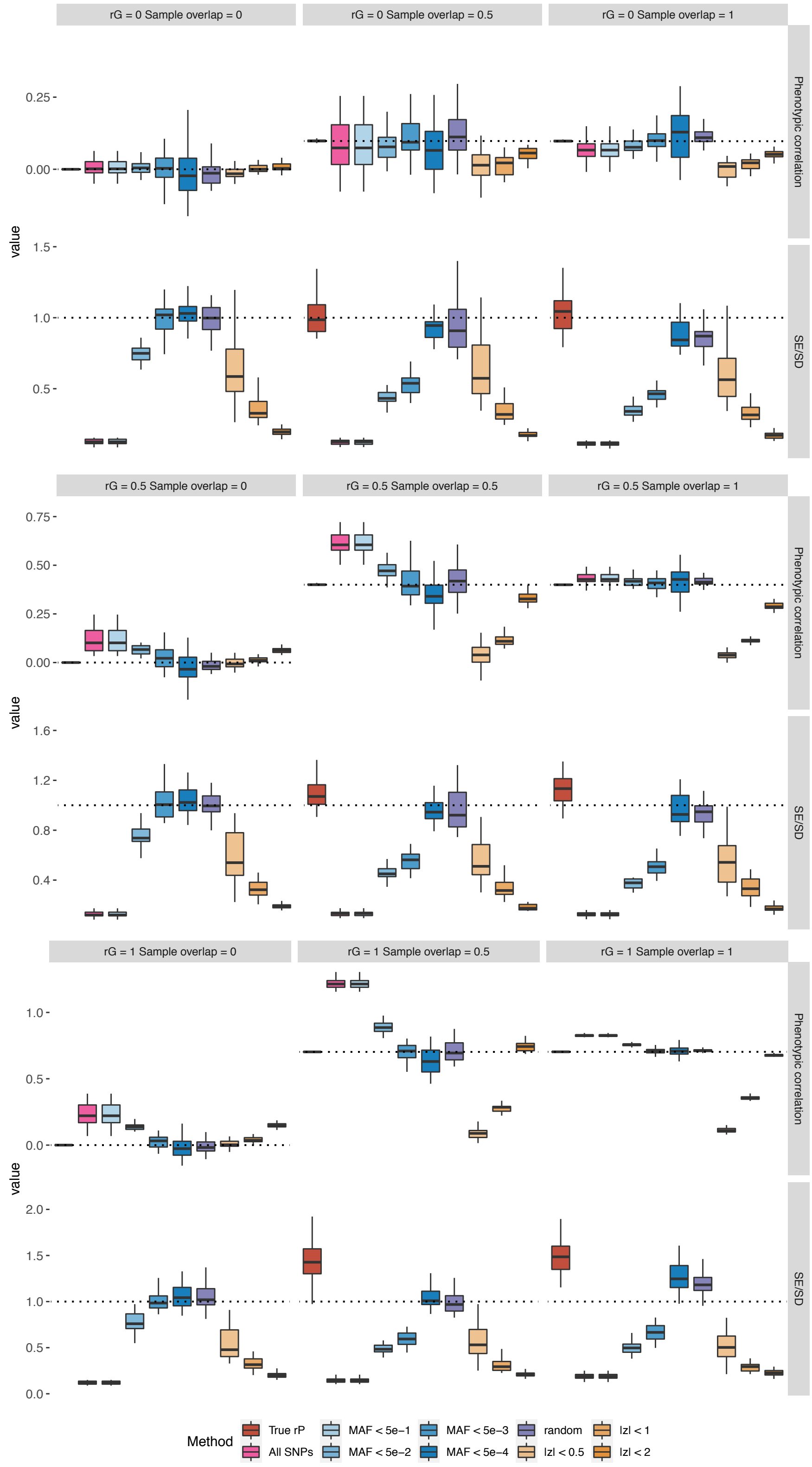
rG = 0 Sample overlap = 0
rG = 0 Sample overlap = 0.5
rG = 0 Sample overlap = 1
rG = 0.5 Sample overlap = 0
rG = 0.5 Sample overlap = 0.5
rG = 0.5 Sample overlap = 1
rG = 1 Sample overlap = 0
rG = 1 Sample overlap = 0.5
rG = 1 Sample overlap = 1
Phenotypic correlation
SE/SD
value
Method
True rP
All SNPs
MAF < 5e−1
MAF < 5e−2
MAF < 5e−3
MAF < 5e−4
random
|z| < 0.5
|z| < 1
|z| < 2

Supplementary Figure 3: Simulations comparing the Z-cut and low-MAF estimators for phenotypic correlation of simulated traits with the heritability of 0.6 based on 10% casual variants. The box plots show the simulation results from 30 replicates, where in each replicate, two phenotypes with heritability of 0.6 were simulated based on 10% randomly selected causal SNPs from the 784,256 genotyped SNPs in the UK Biobank. The true genetic correlation (rG) were set to 0,0.5,1 and the residual correlations were set to 0.25 for each pair of traits. Three scenarios of sample overlap proportions are shown. In the top panels, each phenotype was simulated for 168,000 genomic British individuals. The box plots compare the estimated phenotypic correlations using different estimators. The dash lines represent the true values. The bottom panels compare the estimated standard errors (SE) across the replicates to the standard deviation (SD) of the phenotypic correlation estimates across the 30 replicates, and each phenotype was simulated for 1,000 genomic British individuals. The dash lines at 1 represent that the estimated SE matches the empirical sampling SD. The "random" method represents the estimator based on 500 simulated SNPs with random genotypes and zero genetic effects. The true phenotypic correlation (rP) estimator was based on the individual-level phenotype data. The other estimators were based on the 12,966 SNPs on chromosome 22. MAF: minor allele frequency.

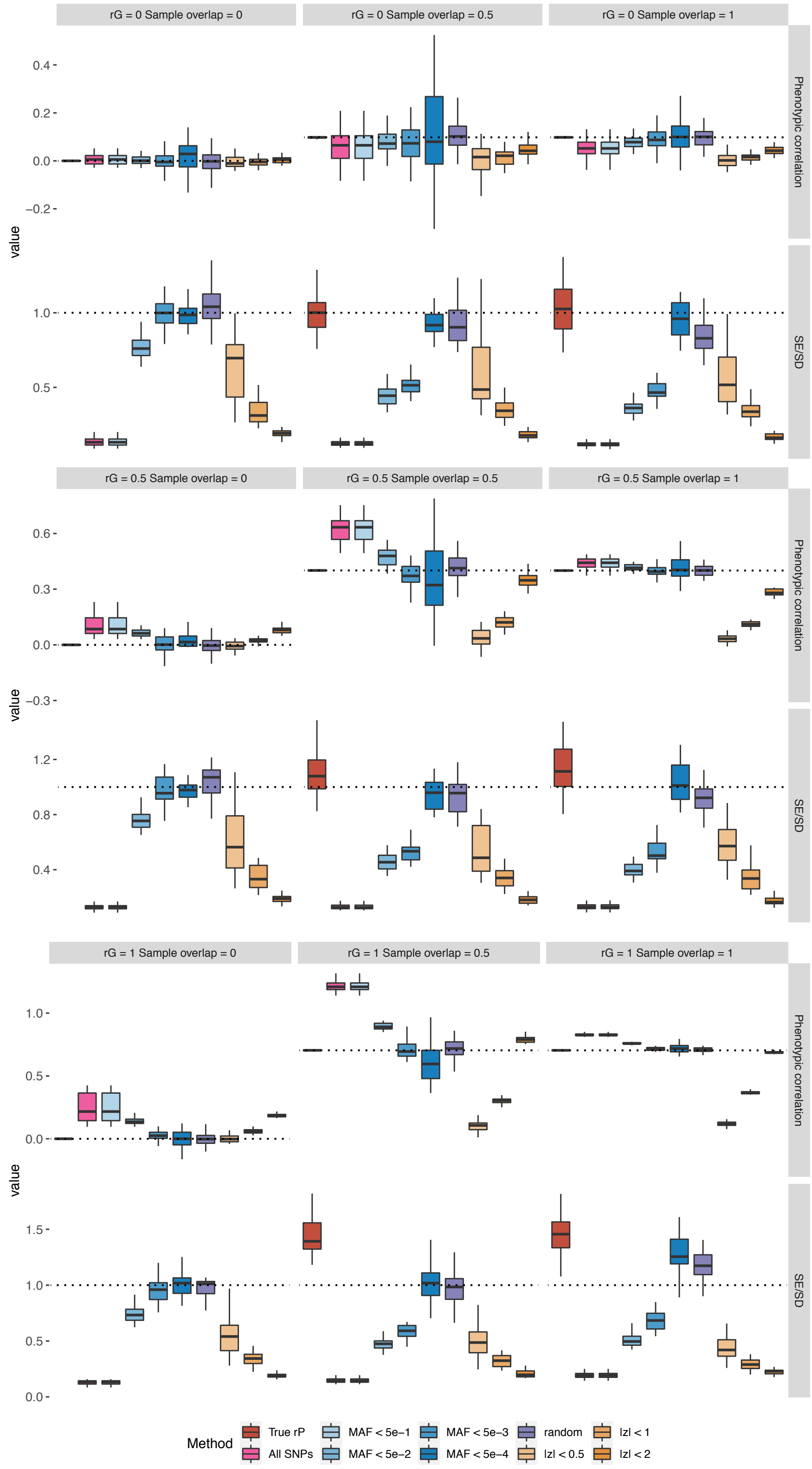

rG = 0 Sample overlap = 0
rG = 0 Sample overlap = 0.5
rG = 0 Sample overlap = 1
rG = 0.5 Sample overlap = 0
rG = 0.5 Sample overlap = 0.5
rG = 0.5 Sample overlap = 1
rG = 1 Sample overlap = 0
rG = 1 Sample overlap = 0.5
rG = 1 Sample overlap = 1
Phenotypic correlation
SE/SD
value
Method
True rP
All SNPs
MAF < 5e−1
MAF < 5e−2
MAF < 5e−3
MAF < 5e−4
random
|z| < 0.5
|z| < 1
|z| < 2

Supplementary Figure 4: Simulations comparing the Z-cut and low-MAF estimators for phenotypic correlation of simulated traits with the heritability of 0.6 based on 100% casual variants. The box plots show the simulation results from 30 replicates, where in each replicate, two phenotypes with heritability of 0.6 were simulated based on 100% causal SNPs from the 784,256 genotyped SNPs in the UK Biobank. The true genetic correlation (rG) were set to 0,0.5,1 and the residual correlations were set to 0.25 for each pair of traits. Three scenarios of sample overlap proportions are shown. In the top panels, each phenotype was simulated for 168,000 genomic British individuals. The box plots compare the estimated phenotypic correlations using different estimators. The dash lines represent the true values. The bottom panels compare the estimated standard errors (SE) across the replicates to the standard deviation (SD) of the phenotypic correlation estimates across the 30 replicates, and each phenotype was simulated for 1,000 genomic British individuals. The dash lines at 1 represent that the estimated SE matches the empirical sampling SD. The "random" method represents the estimator based on 500 simulated SNPs with random genotypes and zero genetic effects. The true phenotypic correlation (rP) estimator was based on the individual-level phenotype data. The other estimators were based on the 12,966 SNPs on chromosome 22. MAF: minor allele frequency.

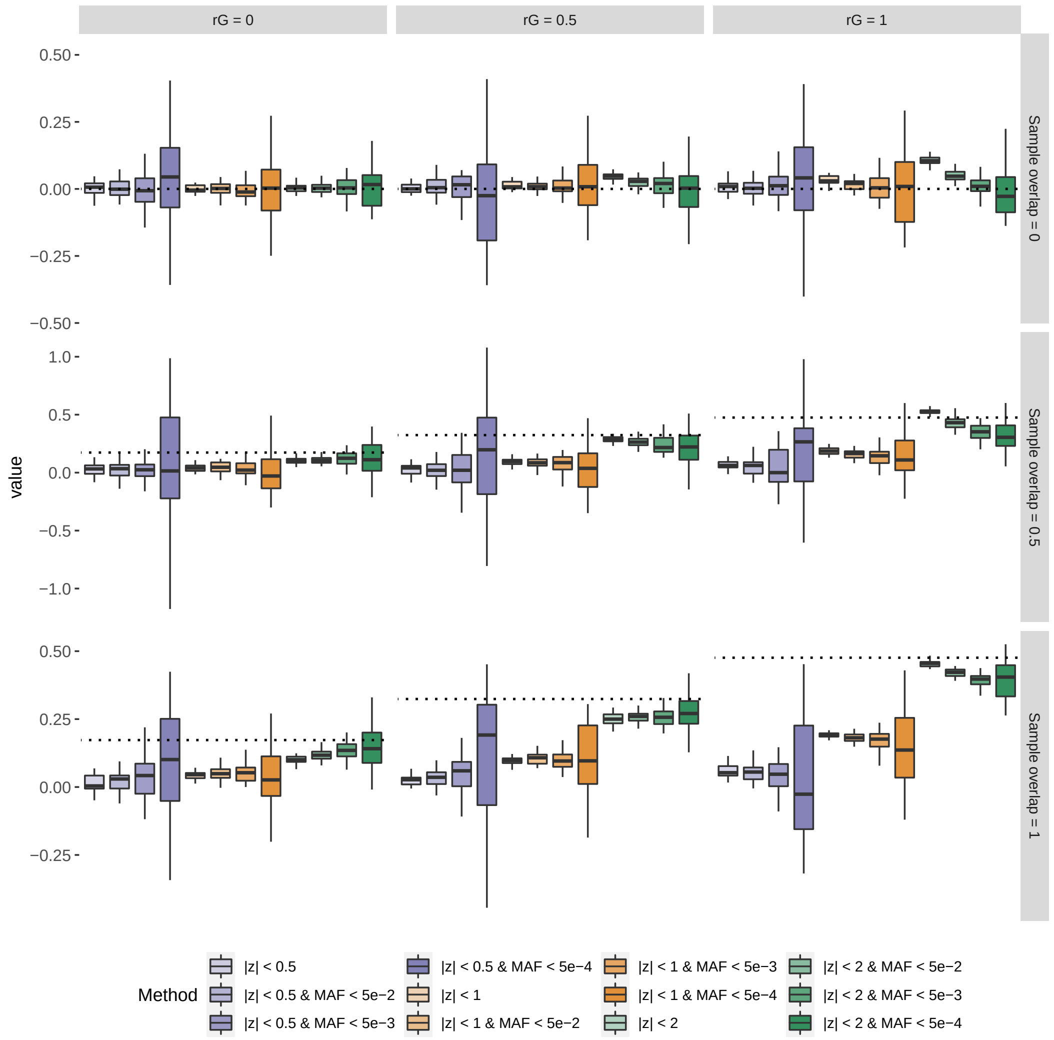

Supplementary Figure 5: Simulations comparing the Z-cut on low-MAF estimators for phenotypic correlation of simulated traits with the heritability of 0.3 based on 10% casual variants The box plots show the simulation results from 30 replicates, where in each replicate, two phenotypes with heritability of 0.3 were simulated based on 10% randomly selected causal SNPs from the 784,256 genotyped SNPs in the UK Biobank. The true genetic correlation (rG) were set to 0,0.5,1 and the residual correlations were set to 0.25 for each pair of traits. Three scenarios of sample overlap proportions are shown. Each phenotype was simulated for 168,000 genomic British individuals. The box plots compare the estimated phenotypic correlations using different estimators. The dash lines represent the true values. The true phenotypic correlation (rP) estimator was based on the individual-level phenotype data. The other estimators were based on the 12,966 SNPs on chromosome 22. MAF: minor allele frequency.

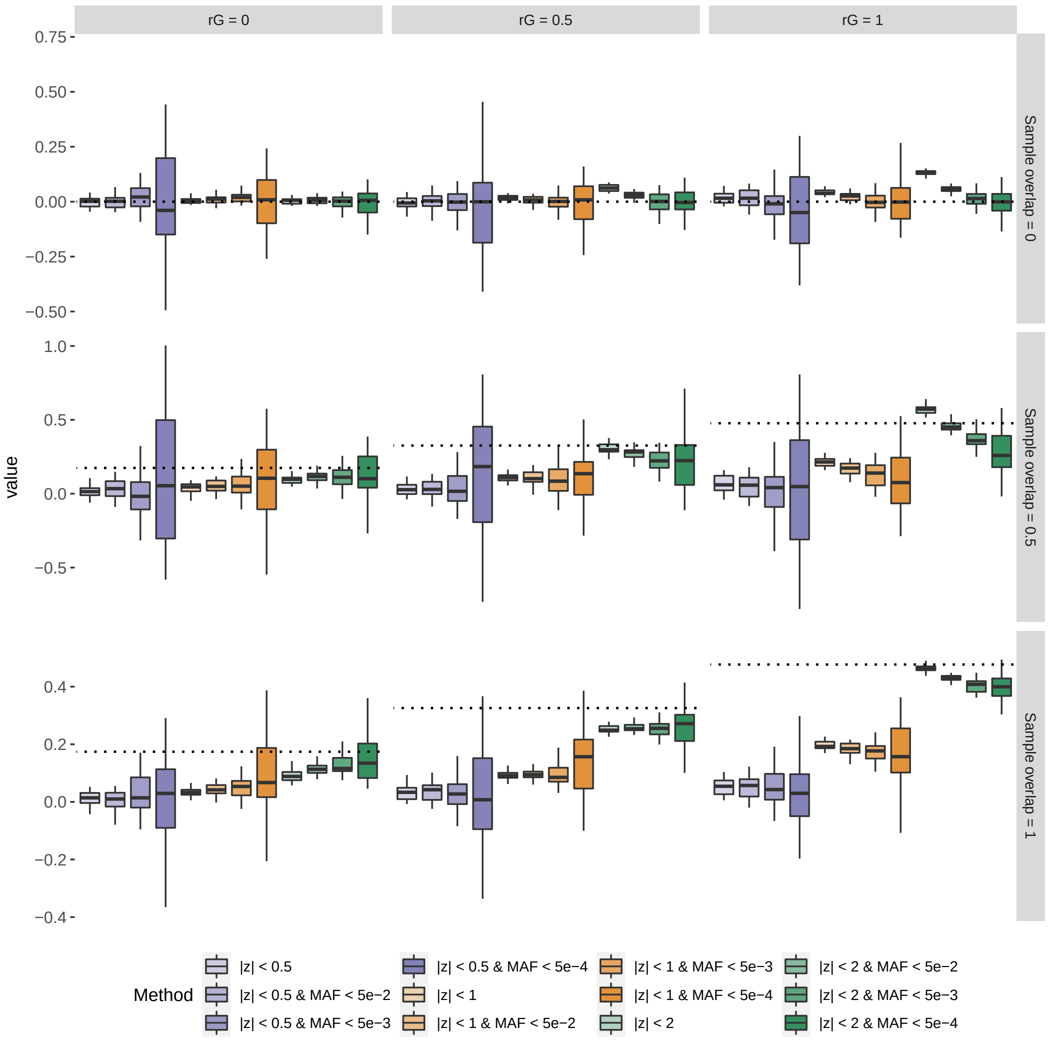

Supplementary Figure 6: Simulations comparing the Z-cut on low-MAF estimators for phenotypic correlation of simulated traits with the heritability of 0.3 based on 100% casual variants The box plots show the simulation results from 30 replicates, where in each replicate, two phenotypes with heritability of 0.3 were simulated based on 100% causal SNPs from the 784,256 genotyped SNPs in the UK Biobank. The true genetic correlation (rG) were set to 0,0.5,1 and the residual correlations were set to 0.25 for each pair of traits. Three scenarios of sample overlap proportions are shown. Each phenotype was simulated for 168,000 genomic British individuals. The box plots compare the estimated phenotypic correlations using different estimators. The dash lines represent the true values. The true phenotypic correlation (rP) estimator was based on the individual-level phenotype data. The other estimators were based on the 12,966 SNPs on chromosome 22. MAF: minor allele frequency.

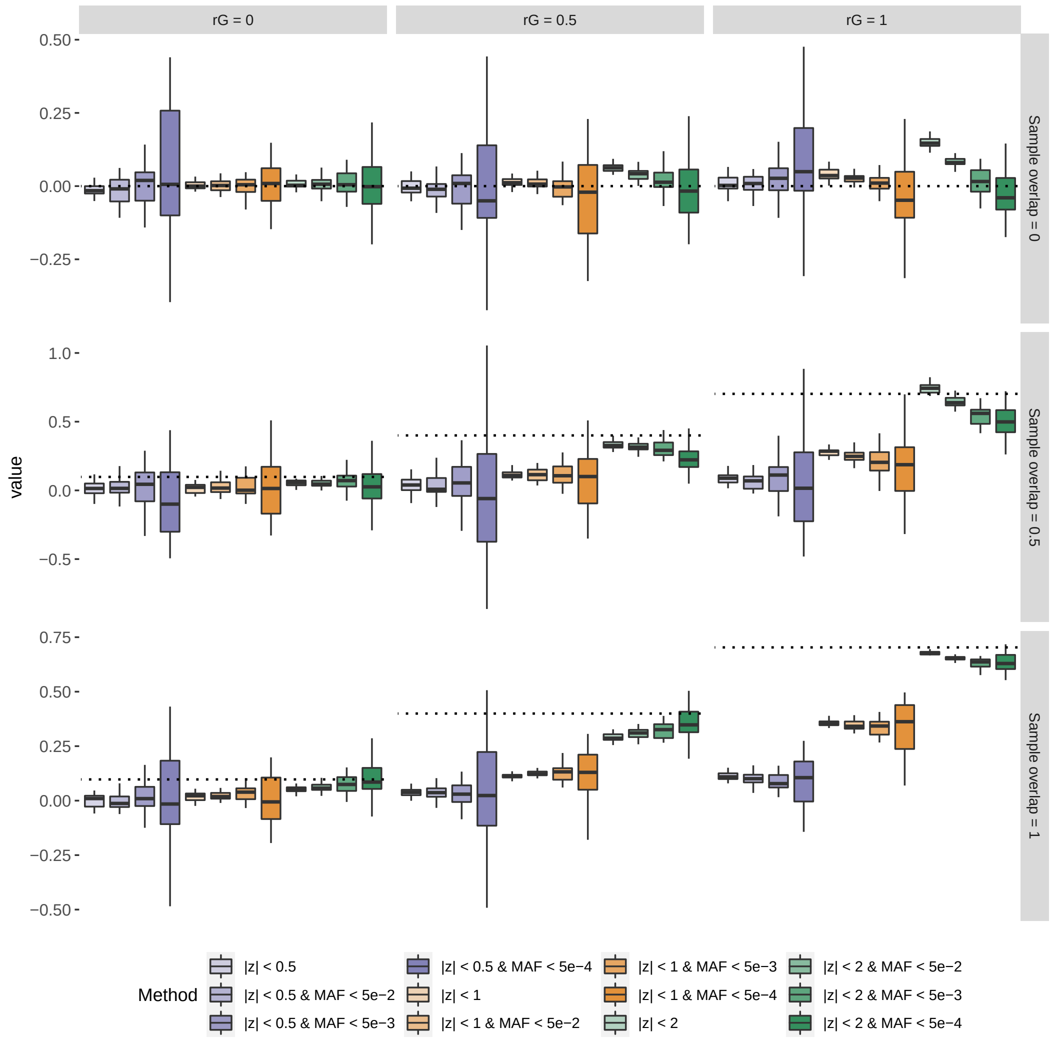

Supplementary Figure 7: Simulations comparing the Z-cut on low-MAF estimators for phenotypic correlation of simulated traits with the heritability of 0.6 based on 10% casual variants The box plots show the simulation results from 30 replicates, where in each replicate, two phenotypes with heritability of 0.6 were simulated based on 10% randomly selected causal SNPs from the 784,256 genotyped SNPs in the UK Biobank. The true genetic correlation (rG) were set to 0,0.5,1 and the residual correlations were set to 0.25 for each pair of traits. Three scenarios of sample overlap proportions are shown. Each phenotype was simulated for 168,000 genomic British individuals. The box plots compare the estimated phenotypic correlations using different estimators. The dash lines represent the true values. The true phenotypic correlation (rP) estimator was based on the individual-level phenotype data. The other estimators were based on the 12,966 SNPs on chromosome 22. MAF: minor allele frequency.

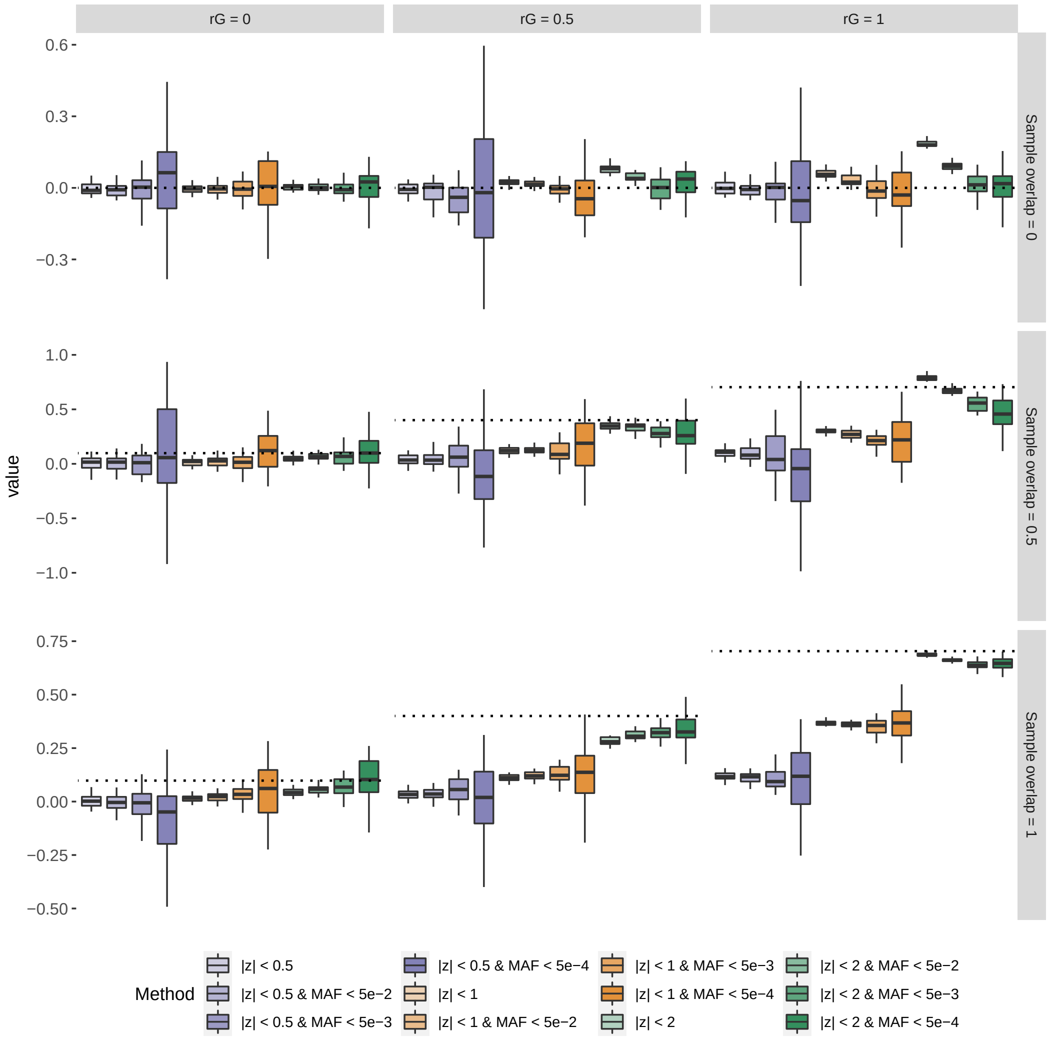

Supplementary Figure 8: Simulations comparing the Z-cut on low-MAF estimators for phenotypic correlation of simulated traits with the heritability of 0.6 based on 100% casual variants The box plots show the simulation results from 30 replicates, where in each replicate, two phenotypes with heritability of 0.3 were simulated based on 100% causal SNPs from the 784,256 genotyped SNPs in the UK Biobank. The true genetic correlation (rG) were set to 0,0.5,1 and the residual correlations were set to 0.25 for each pair of traits. Three scenarios of sample overlap proportions are shown. Each phenotype was simulated for 168,000 genomic British individuals. The box plots compare the estimated phenotypic correlations using different estimators. The dash lines represent the true values. The true phenotypic correlation (rP) estimator was based on the individual-level phenotype data. The other estimators were based on the 12,966 SNPs on chromosome 22. MAF: minor allele frequency.

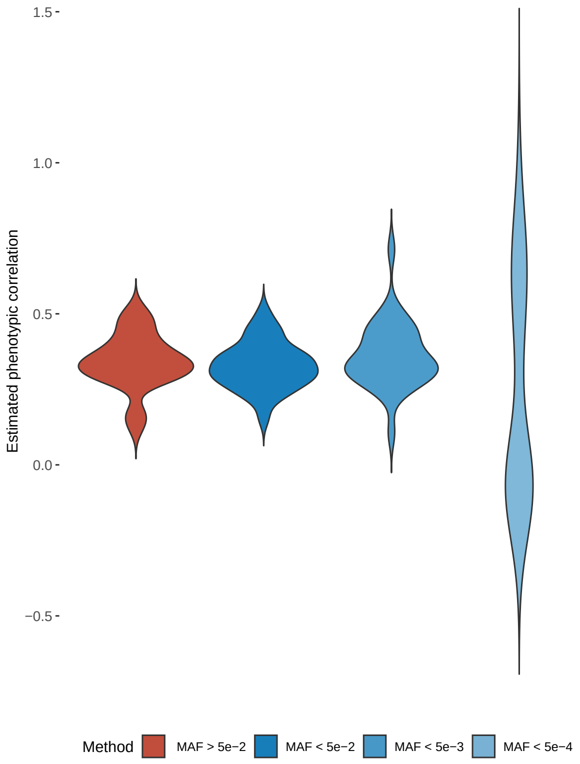

Supplementary Figure 9: Simulations comparing the estimators with different MAF for phenotypic correlation estimation in binary traits. The violin plots show the simulation results from 30 replicates, where in each replicate, the true genetic correlation (rG) were set to 0, and the residual correlations were set to 1 for each pair of traits. 100% sample overlap is considered. Each binary phenotype was simulated for 1,000 genomic British individuals with 10% cases. The box plots compare the estimated phenotypic correlations using different MAF-cutoff estimators, which were based on 1,000 SNPs with MAF $> 5 \times 10^{-2}$, 500 SNPs with MAF $< 5 \times 10^{-2}$, 500 SNPs with MAF $< 5 \times 10^{-3}$, 161 SNPs with MAF $< 5 \times 10^{-4}$.

Supplementary Tables

Supplementary Table 1: Comparison of the mean of phenotypic correlation estimates by the low-MAF and Z-cut estimators. The results are the means of estimated phenotypic correlation summarised from 30 replicates.

[See the Excel File]

Supplementary Table 2: Comparison of the standard deviations of phenotypic correlation estimates by the low-MAF and Z-cut estimators. The results are the standard deviation of estimated phenotypic correlation summarised from 30 replicates.

[See the Excel File]

Supplementary Table 3: The number of SNPs used in phenotypic correlation estimation by the low-MAF and Z-cut estimators.

[See the Excel File]

Supplementary Table 4: Summary-level data of Figure 2. The results were summarised from 100 replicates, where in each replicate, two phenotypes were simulated for 336,000 genomic British individuals. The true phenotypic, genetic, and residual correlations were all set to 0.5. The low-MAF estimates were based on 70,042 SNPs with MAF $< 5e-4$. 1kG ref: LD scores calculated based on the 1000 Genomes reference panel; UKB ref: LD scores calculated based on the UK Biobank reference panel.

	Low-MAF	LDSC (1kG)	LDSC (UKB)
Minimum	0.4865	0.4447	0.4656
25% Quantile	0.4964	0.4719	0.4927
Median	0.4996	0.4887	0.5034
Mean	0.5001	0.4871	0.5040
75% Quantile	0.5035	0.4997	0.5166
Maximum	0.5205	0.5391	0.5498
Variance	3.603e-05	3.626e-04	3.022e-04
